# Unveiling BID: a key biomarker in apoptosis post-intracerebral hemorrhage

**DOI:** 10.3389/fneur.2025.1533558

**Published:** 2025-02-28

**Authors:** Kun Dai, Hong-Rong Zhang, Shuai-Yu Ren, Ming-Pei Zhao, Neng Wang, Hong-Zhi Gao, De-Zhi Kang, Zong-Qing Zheng

**Affiliations:** ^1^Department of Neurosurgery, The First Affiliated Hospital of Soochow University, Suzhou, China; ^2^Department of Neurosurgery, Neurosurgery Research Institute, The First Affiliated Hospital, Fujian Medical University, Fuzhou, China; ^3^Department of Neurosurgery, National Regional Medical Center, Binhai Campus of the First Affiliated Hospital, Fujian Medical University, Fuzhou, China; ^4^Department of Neurosurgery, The Second Affiliated Hospital of Fujian Medical University, Quanzhou, China; ^5^Clinical Center for Molecular Diagnosis and Therapy, The Second Affiliated Hospital of Fujian Medical University, Quanzhou, China; ^6^Fujian Provincial Institutes of Brain Disorders and Brain Sciences, First Affiliated Hospital, Fujian Medical University, Fuzhou, China

**Keywords:** ICH, apoptosis, bioinformatics analysis, microRNA, BID

## Abstract

**Background:**

Apoptosis plays a significant role in secondary brain injury following intracerebral hemorrhage (ICH). Currently, the mechanisms related to cell apoptosis after cerebral hemorrhage are still under investigation.

**Methods:**

We identified differentially expressed genes (DEGs) between human ICH patients and normal individuals from the GEO database and conducted GO and KEGG functional enrichment analyses on these DEGs. We then constructed a PPI network and used the MECDE algorithm to identify key genes potentially involved in apoptosis after ICH. Additionally, we identified miRNAs that might regulate apoptotic genes in an mRNA-miRNA interaction network. Finally, we validated the bioinformatics results in a rat ICH model.

**Results:**

In the human ICH model, 645 DEGs were identified. GO and KEGG analyses indicated that these DEGs are primarily involved in immune response, inflammatory response, and apoptosis. GSEA analysis showed significant enrichment of DEGs in the apoptotic process. By comparing with apoptosis-related genes in the MSigDB database, we identified 110 apoptosis-related genes among the 645 DEGs. Further PPI and MOCDE analyses of these apoptosis-related genes revealed that BID might be a key gene involved in apoptosis after ICH, which was validated within the rat model of ICH. The mRNA-miRNA interactions network construction suggested that miR1225-3p may be an important miRNA involved in regulating BID expression after ICH.

**Conclusion:**

BID plays a critical role in the regulation of apoptosis following intracerebral hemorrhage and serves as a key biomarker in the apoptotic process after hemorrhage.

## 1 Introduction

Intracerebral hemorrhage (ICH) is a hemorrhagic form of stroke characterized by bleeding within the brain tissue (1). This type of stroke occurs when a blood vessel ruptures and blood leaks into the surrounding brain tissue, and it is associated with high morbidity and mortality (2). In 2019, there were approximately 3.41 million new cases of cerebral hemorrhage worldwide, with around 2.89 million resulting in death (3). In the United States, about 30–40% of patients die from cerebral hemorrhage, and those who survive often suffer from multiple comorbidities, severely affecting their quality of life (1). ICH can cause damage to brain cells and tissues, leading to neurological deficits. Recently, numerous studies have been conducted to determine the injury mechanism resulting from ICH (4–6).

Apoptosis, also known as programmed cell death, significantly contributes to the pathogenesis of brain injury resulting from ICH (7). The presence of blood within the brain tissue triggers a cascade of events that can lead to apoptosis. When cerebral hemorrhage occurs, the leading cause is direct damage to brain tissue caused by the hematoma. The release of blood into the brain tissue causes mechanical disruption of cells and blood vessels (1). This disruption can activate apoptotic pathways within the affected cells. The damaged cells may undergo apoptosis as a protective mechanism to prevent further injury and to clear away cells that are beyond repair (8). Following the initial injury, secondary brain damage, characterized by factors such as ischemia, edema, inflammation, and other factors, can exacerbate neurological dysfunction in the brain (9). Apoptosis is often considered a part of the secondary injury phase that follows the initial hemorrhage (10). In response to the primary injury, the brain undergoes a series of events that lead to further cellular damage and eventual cell death. Apoptosis may be a prominent feature of this secondary injury, spreading beyond the initial site of damage. Comprehending the significance of apoptosis in ICH is crucial for formulating therapeutic approaches that target the reduction of cell death and the enhancement of neuroprotection (11).

**Figure 1:**
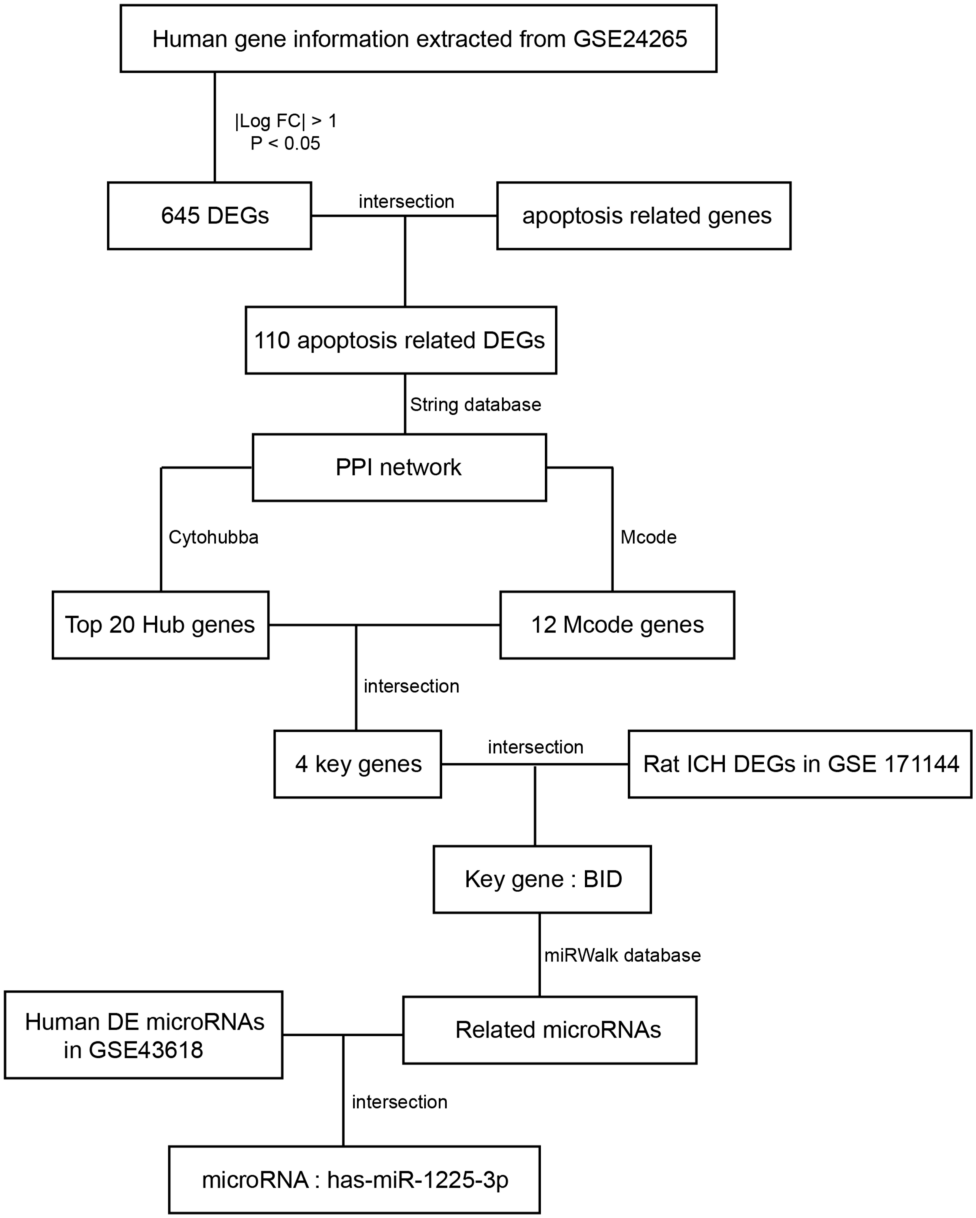
Procedure of the study flowchart.

Our study seeks to investigate and confirm the key genes implicated in apoptosis subsequent to ICH (Figure 1). We conducted an analysis of a human ICH microarray dataset to scrutinize the biological functions of differentially expressed genes (DEGs) in ICH patients compared to healthy controls. Through bioinformatics analysis of DEGs, we identified their association with apoptosis-related genes. Subsequently, in the PPI network analysis, we further identified potential key genes involved in the apoptosis process within the ICH model. Additionally, we investigated miRNAs that may play a role in the modulation of apoptosis through the mRNA/miRNA regulatory network. Finally, we validated the identified key apoptosis genes in the rat ICH model.

## 2 Methods and materials

### 2.1 Experimental animals

All animal experiments were carried out in compliance with the Animal Ethics Procedures and with approval from the Soochow University Animal Ethics Committee. The healthy adult male Sprague Dawley (SD) rats, weighing between 280–320 grams and aged 8–12 weeks, were sourced from the Animal Center of the Chinese Academy of Sciences (Shanghai, China). Throughout the experiment, the rats were subjected to feeding, utilization, and surgical procedures in strict compliance with the established regulations for the ethical treatment of laboratory animals. A total of 16 rats were involved, and their grouping followed randomized, controlled, and double-blind principles. The evaluation of animals within each group was carried out using a double-blind protocol, ensuring that the investigators were unaware of both the animals’ serial numbers and their respective experimental groups. Every effort was made to minimize the rats’ suffering throughout the experiments. Data obtained from studies on animals have been documented in accordance with the Animal Research: Reporting of *In Vivo* Experiments (ARRIVE) guidelines (12).

### 2.2 Establishing the ICH model *in vivo*

The *in vivo* ICH model of rats was established as described previously (13). In briefly, the rats were anesthetized with 3% isoflurane and subsequently positioned in a stereotactic device. Utilize an electric drill to bore a hole into the right parietal bone of the rat’s skull, precisely located 2 mm posterior to the bregma and 3.5 mm lateral to the sagittal suture. Insert the micro syringe vertically into the brain tissue, reaching the basal ganglia area at a depth of 5.5 mm. Administer a slow injection (10–15 μL/min) of 100 μL autologous blood and allow it to stand for 5 min to avoid blood reflux. The Sham group also underwent craniotomy, but no blood was injected into the brain.

### 2.3 Western blot

Western blot analysis was conducted following the standard protocol as described in reference (14). To ensure uniform loading, protein concentrations were quantified using the bicinchoninic acid (BCA) protein assay. Protein samples, each containing 30 μg, were loaded per lane onto a 10% SDS-polyacrylamide gel. Post-electrophoresis, the proteins were transferred onto a nitrocellulose membrane (Millipore) and incubated with primary antibodies overnight at 4°C. Subsequently, the membrane was incubated with secondary antibodies for 1 h. The protein bands on the membrane were visualized using an enhanced chemiluminescence detection kit (Thermo Fisher Scientific). Quantitative analysis of the images was performed using ImageJ software.

### 2.4 Immunofluorescence analysis

The brain tissue of SD rats was initially fixed in a 4% paraformaldehyde solution, followed by paraffin embedding and sectioning into 4 μm slices. The dewaxing process was conducted using xylene and ethanol, after which antigen retrieval was performed using citric acid. Permeabilization was achieved through treatment with 0.5% Triton X-100. The slices were then incubated with a primary antibody overnight at 4°C, followed by a 1-h incubation with an appropriate secondary antibody. Finally, the sections were counterstained with DAPI and examined under a fluorescence microscope.

### 2.5 TUNEL staining

TUNEL assay was carried out with the *In situ* TUNEL kit (Roche). After dewaxing and dehydration, sections underwent antigen repair with citric acid. The sections were subsequently treated with 0.5% Triton X-100 to induce permeabilization. Following this, they were incubated with TUNEL staining for 60 min at 37°C and then washed three times with PBS. After overnight incubation with primary antibodies at 4°C, the sections were exposed to suitable secondary antibodies for 1 h then counterstained with DAPI before being examined using a fluorescence microscope.

### 2.6 Identification DEGs in ICH datasets

This study utilizes three datasets from the Gene Expression Omnibus (GEO) database,^1^ specifically GSE24265, GSE171144, and GSE43618. The GSE24265 dataset includes 11 human brain samples collected from four deceased patients who had experienced intracerebral hemorrhage. These samples encompassed perihematomal tissue, along with the corresponding contralateral white and grey matter. Meanwhile, the GSE171144 dataset elucidates alterations in gene expression between perihematomal tissues and normal brain tissues in rats, encompassing three cases each in both the ICH and sham groups. In GSE43618, differences in plasma miRNA expression were observed between patients with cerebral hemorrhage and healthy controls, each group consists of two cases. The DEGs were analyzed through the GEO2R online analysis tool, with |log2 (foldchange)| and *p*-value set as parameters. Subsequently, the outcomes were graphically represented by employing the R 4.3.1 software in conjunction with the ggplot2 package.

### 2.7 Differentially expressed apoptosis-related genes

A sum of 1,965 genes linked to human apoptosis were obtained from the Molecular Signatures Database (MSigDB).^2^ Upon screening of DEGs from GSE24265 and human apoptosis-related genes, 110 differentially expressed human apoptosis-related genes were identified.

### 2.8 Functional annotation

To elucidate the biological functions of DEGs, we conducted Gene Ontology (GO) annotation and Kyoto Encyclopedia of Genes and Genomes (KEGG) pathway enrichment analyses. Furthermore, we utilized Gene Set Enrichment Analysis (GSEA) to examine the associations between DEG enrichment and various gene sets from the Molecular Signatures Database (MSigDB Collections) (see text footnote 2). The significance threshold for all enrichment analyses was set at an adjusted *p*-value (*p*.adj) of less than 0.05. The analyses were utilizing R version 3.6.3 in conjunction with “clusterProfiler” package, and the results were visualized using the “ggplot2” package.

### 2.9 Construction of protein–protein interaction networks and analysis of hub genes

Data on protein–protein interactions (PPI) among genes was extracted from the STRING database,^3^ using confidence scores of ≥0.4. Cytoscape software was employed to facilitate the visualization of results, culminating in the construction of the gene coexpression network. Subsequently, the MCODE algorithm within Cytoscape was applied to identify densely connected gene clusters within PPI network. The CytoHubba plugin within Cytoscape software platform was then utilized to identify the top 20 hub genes from the DEGs using node degree ranking algorithms. From the intersection of these 20 hub genes and 12 MCODE-identified genes, four key genes were selected for additional validation concerning apoptosis following ICH.

### 2.10 Key genes identification and miRNA characterization

The key gene, BID, was discerned through the convergence of four key genes and DEGs associated with ICH model of rat. The miRWalk database^4^ was employed to forecast microRNAs (miRNAs) and to construct gene-miRNA interaction networks. To ensure the precision of the results, an interaction score threshold of at least 1 and an interaction position within the 5′ untranslated region (5UTR) were established. We selected the top 30 miRNAs based on their interaction scores. The potential miRNAs targeting BID were validated in the plasma of patients with intracerebral hemorrhage (GSE43618), with a particular focus on miRNAs related to human apoptosis.

### 2.11 Statistical analysis

Statistical analyses were conducted utilizing GraphPad 9.0.0 (Prism) software, with the mean ± SD values reported for all data. Initial assessments involved checking for normal distribution. Following this, the results were subjected to relevant statistical analyses. The comparison between two groups was conducted through Student’s *t*-test, with statistical significance set at *p* < 0.05.

## 3 Result

### 3.1 Identification of DEGs in human ICH

**Figure 2:**
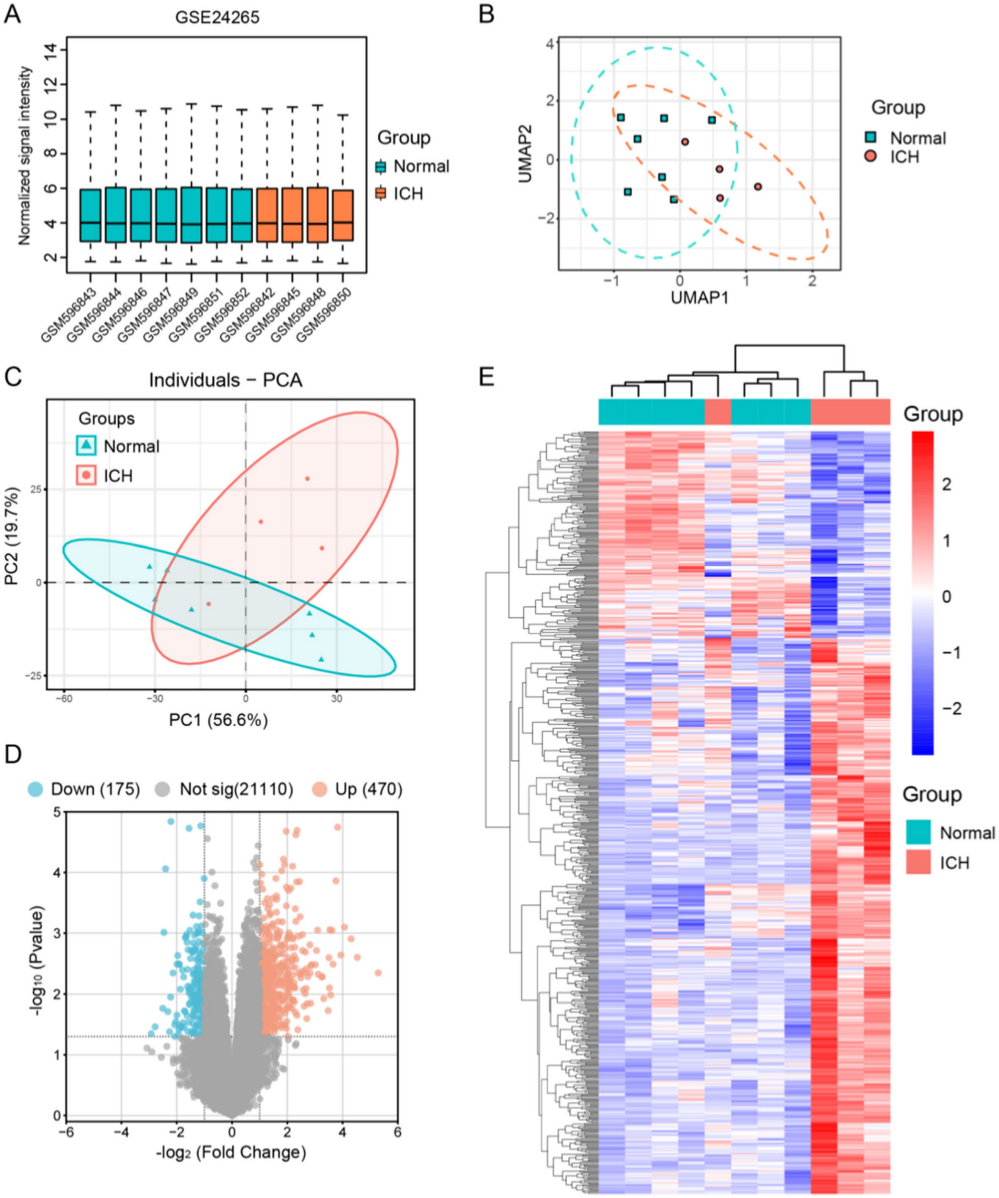
Identification of the differential genes between human ICH patients and normal brain tissue samples in GSE24265. **(A)** Normalization of signal intensity for 11 samples in GSE24265. **(B)** UMAP dimensionality reduction and visualization of the data. **(C)** Principal component analysis (PCA) results of the 11 samples. **(D)** Visualization of differential genes using a volcano plot, showing 175 genes downregulated and 470 upregulated in ICH. **(E)** Clustering heatmap of 645 DEGs.

We extracted data from the GSE24265 microarray dataset for seven healthy individuals and four intracerebral hemorrhage patients. Before proceeding with further data processing, we first performed data normalization (Figure 2A). PCA and UMAP were utilized to perform dimensionality reduction analysis on the variability of gene expression across various samples (Figures 2B,C). By comparing these two sets of data, 645 DEGs were identified, among which 470 genes were upregulated and 175 genes were downregulated after ICH (Figure 2D). Subsequently, we performed clustering analysis on the DEGs and displayed a heatmap (Figure 2E).

### 3.2 Functional enrichment analysis

**Figure 3:**
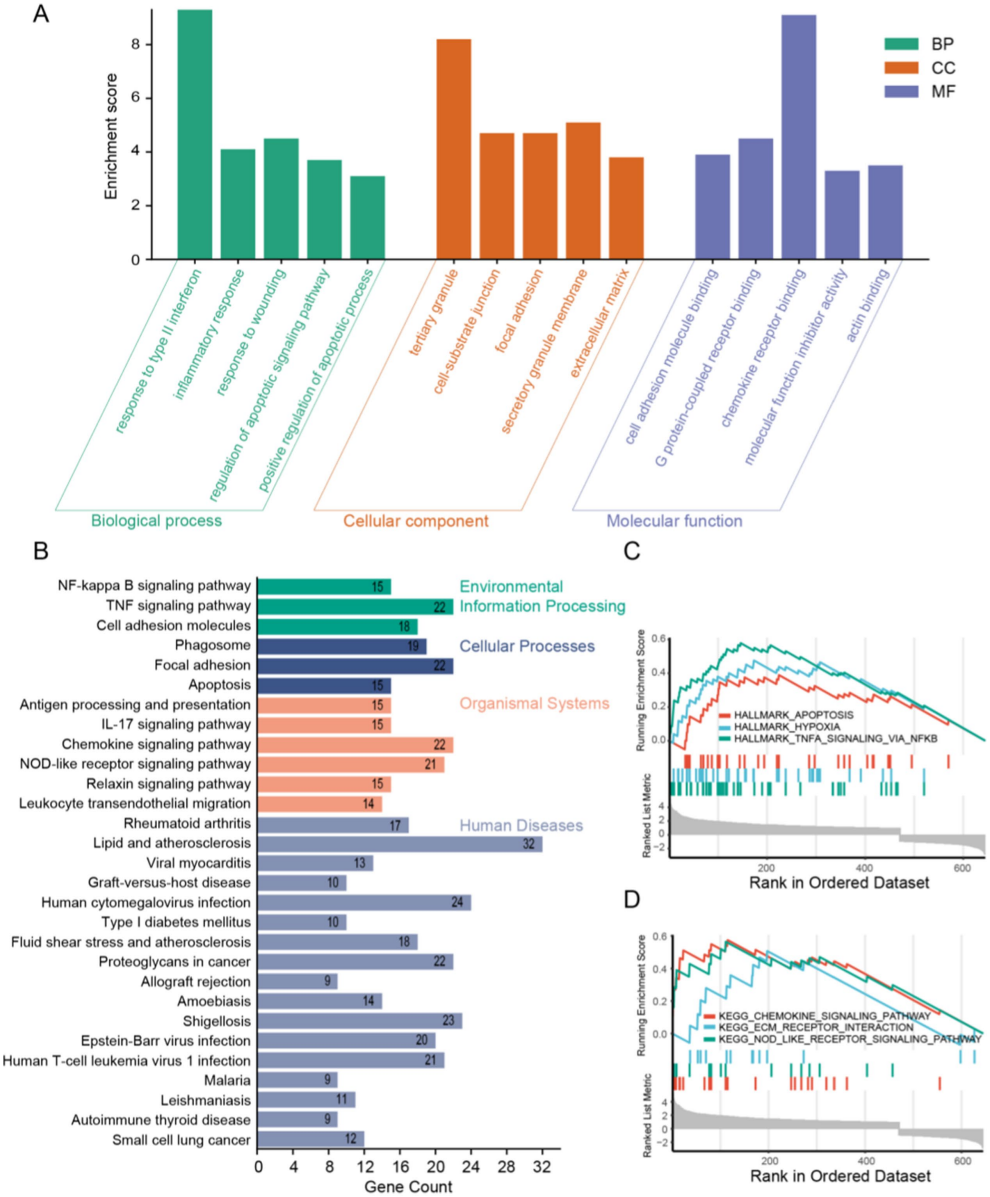
Functional enrichment analysis of DEGs. **(A)** GO analysis results showing the top five in BP, CC, and MF. **(B)** KEGG analysis results and visualization. **(C,D)** GSEA analysis results of DEGs.

GO and KEGG, two bioinformatics resources, were adopted for functional annotation and pathway analysis of DEGs. Subsequently, we identified the top five enriched terms in biological process (BP), cellular component (CC), and molecular function (MF), and visualized the results (Figure 3A). The BP primarily implicated in DEGs include immune response, inflammatory response, and apoptosis. The CC of DEGs were predominantly enriched in tertiary granules, cell-substrate junctions, and focal adhesions. Functionally, DEGs were chiefly associated with cell adhesion molecule binding, G protein-coupled receptor binding, and chemokine receptor binding. According to the KEGG analysis, DEGs were linked to the NF-kappa B signaling pathway, the TNF signaling pathway, among others. For more detailed information, please refer to Figure 3B. Furthermore, gene set enrichment analysis of the DEGs revealed potential associations with apoptosis, hypoxia, and the TNFA signaling pathway (Figures 3C,D).

### 3.3 PPI network construction and hub genes identification

**Figure 4:**
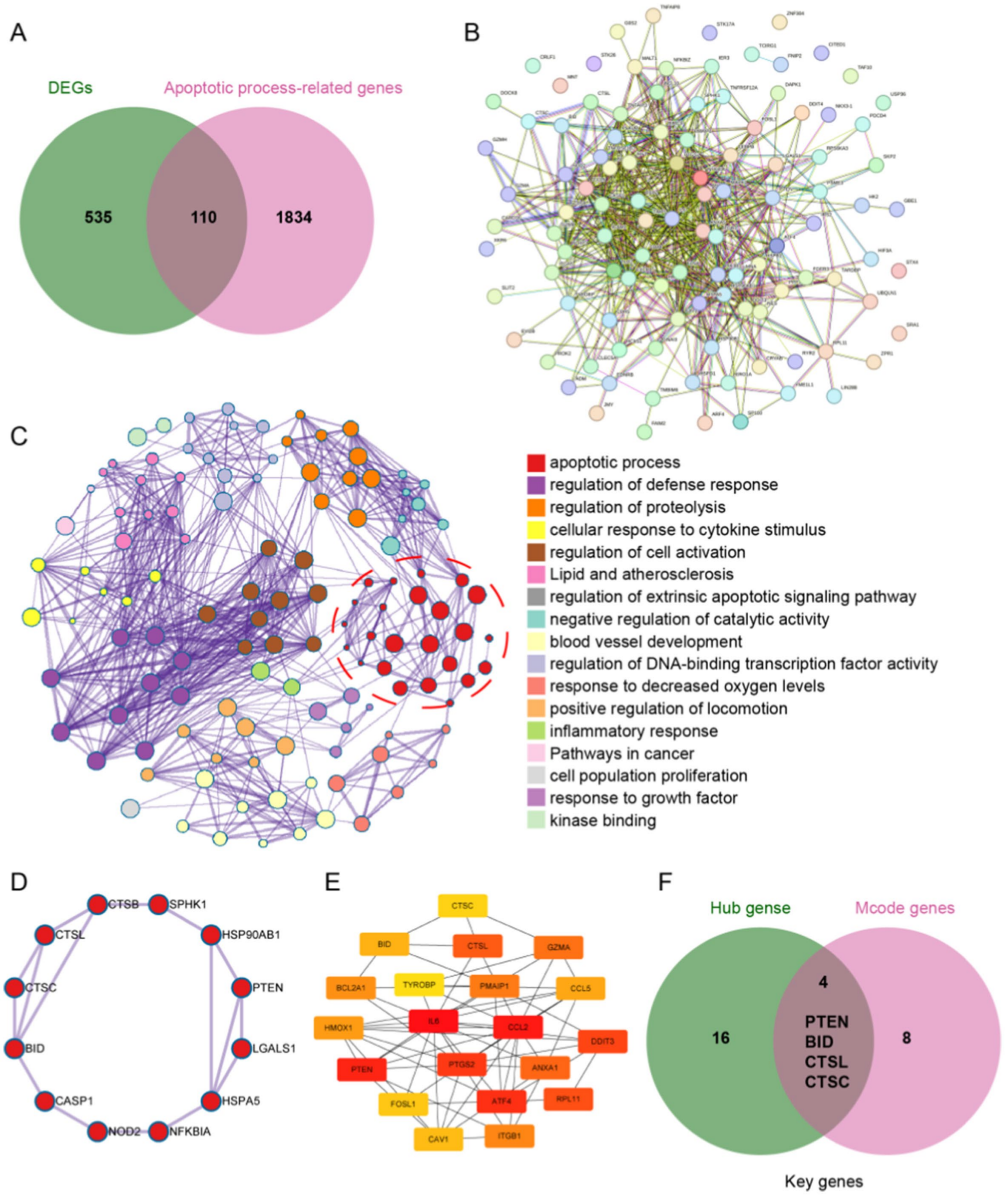
Identification of apoptosis-related genes. **(A)** The intersection of DEGs and apoptosis-related genes revealed 110 apoptosis-related genes after ICH. **(B)** Protein–protein interaction network of the 110 apoptosis-related genes. **(C)** PPI analysis of apoptosis-related genes, clustered by function. **(D)** Identification of 12 MCODE genes by MCODE analysis. **(E)** Top 20 hub genes in the PPI were identified by using Cytoscape software. **(F)** The intersection of the 12 MCODE genes and the top 20 hub genes yielded four key genes.

To identify potential apoptosis-related genes among the 645 DEGs, we first retrieved 1,944 apoptosis-related genes from the MSigDB database and intersected them with the DEGs, resulting in 110 apoptosis-related genes (Figure 4A). Next, we constructed a PPI network using the String database for these apoptosis-related genes and classified them based on protein function (Figures 4B,C). We then analyzed these apoptosis-related genes using the MCODE method, identifying 12 MCODE genes (Figure 4D). Using Cytoscape software, we analyzed the PPI network and selected the top 20 HUB genes through cytoHubba (Figure 4E). Finally, we intersected the MCODE genes and HUB genes, ultimately identifying four key genes: PTEN, BID, CTSL, and CTSC (Figure 4F). These four genes were all upregulated after ICH, possibly playing a pivotal role in apoptosis cascade following ICH.

### 3.4 Key genes identification and miRNA characterization

**Figure 5:**
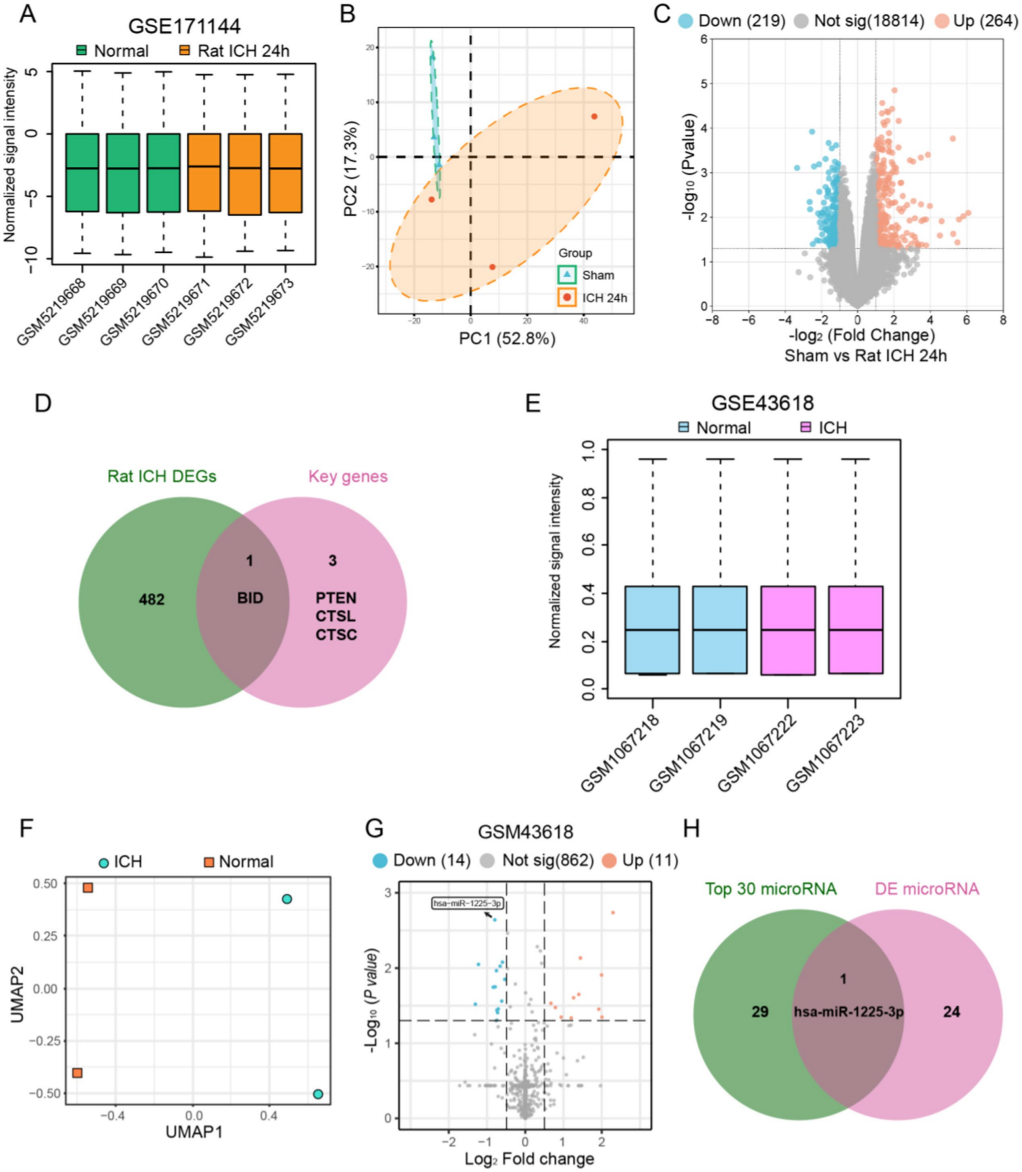
Identification of key genes in the apoptosis process after ICH and determination of the regulating microRNA. **(A)** Normalization of signal intensity for six rats ICH samples in GSE171144. **(B)** PCA results of the six rats ICH samples in GSE171144. **(C)** Visualization of differential genes in GSE171144 using a volcano plot. **(D)** The intersection of rat ICH DEGs and four key genes revealed apoptosis-related genes after ICH. **(E)** Normalization of signal intensity for four human ICH samples in GSE43618. **(F)** UMAP dimensionality reduction and visualization of the GSE43618. **(G)** Visualization of differential microRNAs in GSE43618 using a volcano plot. **(H)** The intersection of top 30 microRNAs and DE mircoRNAs revealed apoptosis-related microRNA after ICH.

For the purpose of further validating the key genes that have been identified, we conducted additional verification in a rat ICH model. First, we normalized and performed principal component analysis on the rat ICH model microarray dataset (GSE171144) (Figures 5A,B). Then, we extracted data from the ICH-24 h and SHAM groups within the dataset, identifying 483 DEGs between the two groups (Figure 5C). Next, we intersected the rat DEGs with the key genes and found that BID is a common DEG in both rat and human specimens (Figure 5D).

We extracted data from human plasma samples (GSE43618), normalizing and performing dimensionality reduction analysis on each group of data before processing (Figures 5E,F). We then identified 25 differentially expressed miRNAs between the ICH group and the Normal group (Figure 5G). From the miRWalk database, we obtained the relevant miRNAs that regulate BID and took the top 30 to find the intersection with the differentially expressed miRNAs, resulting in miR1225-3p (Figure 5H). We found that miR1225-3p was downregulated after ICH, suggesting that the downregulation of miR1225-3p may be associated with the upregulation of BID.

### 3.5 Validation of BID in rat ICH model

**Figure 6:**
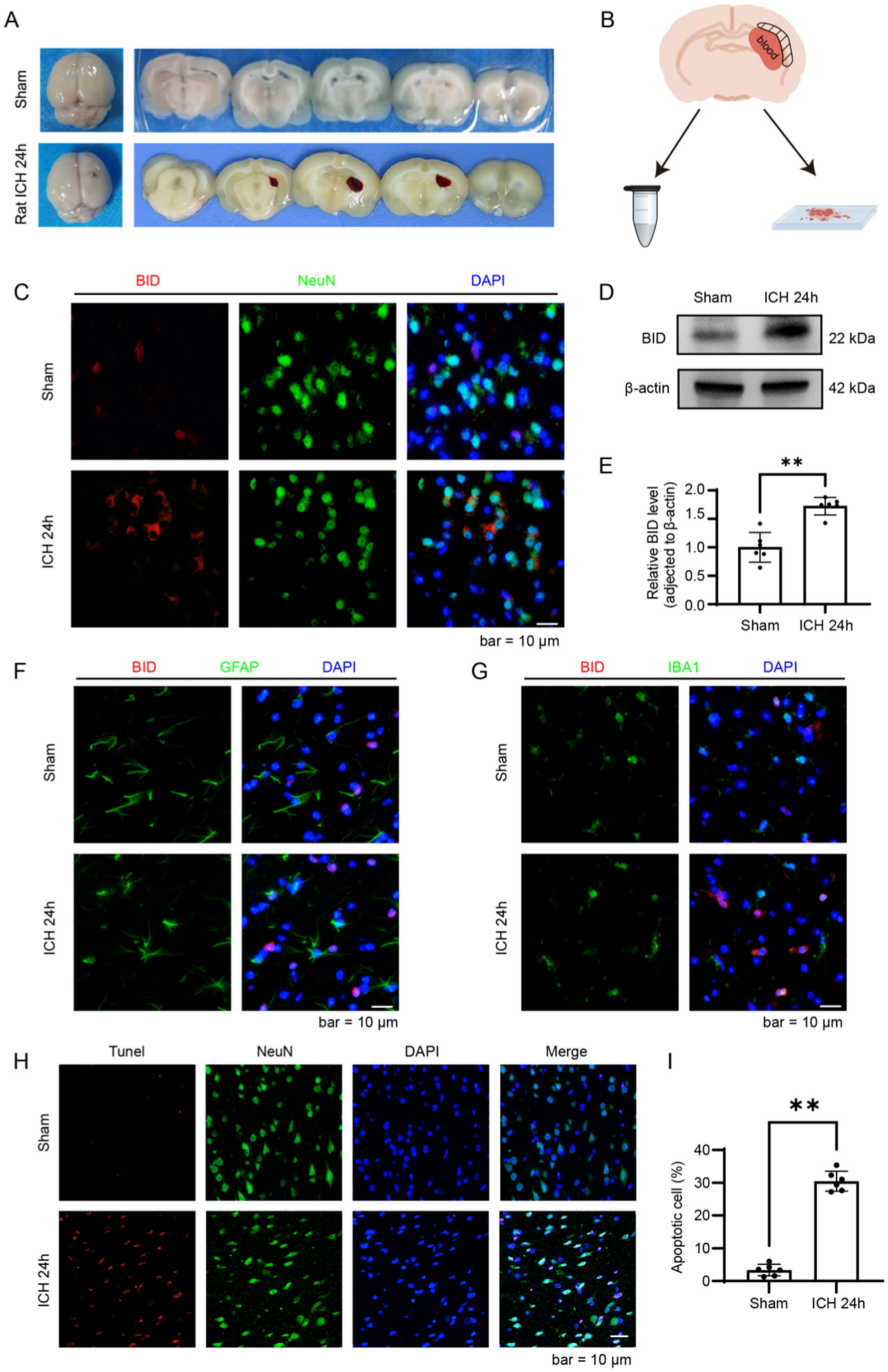
Validation of BID in a rat ICH model. **(A)** Brain tissue sections of rats in the ICH group and Sham group. In the ICH group, a hematoma was observed in the right basal ganglia of rats, while no bleeding was observed in the Sham group. **(B)** Diagram of brain tissue sampling. Take brain tissue from the peripheral area of the hematoma (i.e., the shadowed area) and prepare it as paraffin sections or grind and lyse it to prepare protein samples. **(C)** Immunofluorescence staining results of BID in brain tissues of rats with ICH and in the Sham group. NeuN (green) was used as a marker for neurons. **(D,E)** Western blot results of BID after intracerebral hemorrhage. β-actin was used as a loading control. **(C)** Presents a quantitative assessment of the BID level. Sham versus SAH, *p* < 0.01, *n* = 6. **(F)** Colocalization of BID with GFAP in immunofluorescence, where GFAP served as a marker for astrocytes. **(G)** Colocalization of BID with IBA1 in immunofluorescence, where IBA1 served as a marker for microglia. **(H,I)** TUNEL staining results for the rat ICH group and the Sham group. **(G)** Presents a quantitative assessment of the apoptotic rates. Sham versus SAH, *p* < 0.01, *n* = 6.

We validated the results obtained from bioinformatics analysis in a rat ICH model. Samples were collected 24 h after the completion of the SD rat ICH model, with the sampling area being the brain tissue surrounding the hematoma (Figures 6A,B). In the immunofluorescence results, we found that the fluorescence intensity of BID in the ICH 24 h group was significantly higher compared to the Sham group (Figure 6C). Similar results were observed in the western blot experiment, where the BID protein expression exhibited a marked elevation in the ICH 24 h group compared to the Sham group (Figures 6D,E). Subsequently, we performed immunostaining for BID with GFAP and IBA1, and found that BID had a small amount of colocalization with both (Figures 6F,G). This indicates that BID is primarily expressed in neuronal cells, with relatively less expression in astrocytes and microglia. Finally, we conducted TUNEL staining on the rat ICH model and found that the TUNEL-positive rate in the ICH 24 h group was significantly higher than in the Sham group, indicating a marked increase in neuronal apoptosis 24 h after ICH (Figures 6H,I).

## 4 Discussion

ICH indeed represents a critical form of stroke with high morbidity and mortality rates. It occurs when a blood vessel within the brain suddenly ruptures, allowing blood to leak into the brain tissue, leading to direct damage to brain cells and secondary injury through mechanisms such as inflammation, edema, and increased intracranial pressure. One of the significant pathways contributing to secondary brain injury post-ICH is the apoptosis of neurons (15). Apoptosis is widely recognized as a key driver of the pathological manifestations associated with ICH. This process is regulated by both extrinsic and intrinsic pathways, leading to the activation of various proteins (15). Among these are the Bcl-2 family proteins, including Bax, Bad, Bid, Bcl-XS, and Bcl-XL, as well as a range of caspases categorized into initiator, effector, and inflammatory caspases (16). These proteins and caspases interact through a well-orchestrated cascade of events, ultimately culminating in cellular disintegration (17). In the meantime, apoptosis represents a systematic series of events in which cells consciously initiate their own demolition (8). This pivotal mechanism is instrumental in sustaining cellular equilibrium and fostering growth in robust tissues. However, in the context of ICH, excessive or inappropriate activation of apoptosis contributes to the loss of neurons and other brain cells, exacerbating brain injury (7, 11). Currently, the role of apoptosis in ICH is still under further study.

In pursuit of identifying genes involved in the pathway associated with apoptosis following ICH, we initially employed bioinformatics methods to delineate the differential genes between hemorrhagic brain patients and normal human brains, amounting to 645 in total. Subsequently, an in-depth functional enrichment analysis of these differential genes revealed the participation of 110 genes in apoptosis. Ultimately, through the execution of PPI network analysis and HUB gene screening on these 110 genes, we unearthed four genes implicated in brain cell apoptosis: PTEN, BID, CTSL, and CTSC. PTEN is recognized as a dual-specificity protein phosphatase, which plays a significant role in numerous biological processes encompassing apoptosis, lipid metabolism, and neurogenesis, among others (18). PTEN, as a tumor suppressor gene, has been extensively researched in various forms of cancer (18). Additionally, abundant studies have corroborated the involvement of PTEN in neuronal protection following ICH (19). Both CTSC (cathepsin C) and CTSL (cathepsin L) are members of the lysosomal protease cathepsin family (20). These enzymes play critical roles in protein degradation within lysosomes, and are implicated in various cellular processes, inclusive of apoptosis (21). Despite this, research focusing on their implications in ICH remains conspicuously scant. BID represents a critical pro-apoptotic constituent within the extensively studied Bcl-2 protein family, renowned for its fundamental involvement in apoptosis regulation (22).

At present, there are no studies on the biological effects of BID after ICH, thereby prompting us to undertake pertinent experimental investigations. In our exploration conducted within a rat model of ICH, we examined the association between BID and apoptosis. We observed that BID was extensively distributed among neurons, astrocytes, and microglia within the cerebral tissue of rats. Post-ICH, there was a notable augmentation in the protein expression levels of BID. Concurrently, an increased incidence of neuronal apoptosis was observed following ICH. Thus, we hypothesize that BID is implicated in the neuronal apoptosis observed subsequent to ICH.

BID belongs to the BCL-2 family, which participates in the intrinsic apoptosis pathway by mediating the permeability of the mitochondrial outer membrane. This family is characterized by the common expression of Bcl-2 homology (BH) domains (16). Differences in the number and types of BH domains distinguish the proteins in this family into pro-apoptotic proteins (e.g., BID, BAX, BAD) and anti-apoptotic proteins (e.g., BCL-2, BCL-XL) (23, 24). BID also plays a certain role in extrinsic apoptosis. The extrinsic pathway is initiated by the binding of extracellular death ligands to their respective death receptors on the cell surface, leading to the activation of caspase-8 (25). Once activated, caspase-8 can cleave BID to generate a truncated form known as tBID (26). tBID is highly potent and can translocate to the mitochondria, where it interacts with and activates Bax and Bak, pro-apoptotic proteins that permeabilize the mitochondrial outer membrane. The permeabilization process results in the release of cytochrome c and other pro-apoptotic factors into the cytosol, which is a crucial step in the intrinsic pathway of apoptosis (27).

In the context of neurological diseases, research on the mechanisms of apoptosis involving BID has made certain progress. The expression of BID in neurons can be observed to increase 90 min after MCAO in mice (28). Cortical neurons in mice undergo BID-dependent apoptosis mediated by caspase-1 after stimulation with nigericin (29). In the study of neuronal apoptosis caused by epilepsy, researchers found that status epilepticus triggers BID activation, conversion to tBID, and subsequent translocation into mitochondria. However, BID gene knockout in mice did not reduce apoptosis (30). In the disease model of cerebral hemorrhage, there are currently no studies on the involvement of BID in the apoptosis mechanism.

We also recognize the limitations of this study. First, although we validated the expression of BID and its relationship with apoptosis in a rat intracerebral hemorrhage model, we did not delve into its underlying mechanisms. Second, the limited sample size of human intracerebral hemorrhage data in the GEO database may introduce bias in the bioinformatics results. Lastly, our preliminary conclusions are based on bioinformatics studies of human brain samples. Due to ethical constraints, we can only validate these findings in animal models, and species differences may lead to discrepancies in the validation results.

BID serves as a critical amplifier of apoptotic signals, ensuring the effective execution of apoptosis in response to various cellular stresses (31). Dysregulation of BID’s function or expression has been implicated in various diseases, where its pathways might be targeted for therapeutic intervention.

## Data Availability

The original contributions presented in the study are included in the article/supplementary material, further inquiries can be directed to the corresponding author.

## Footnotes

^1^ https://www.ncbi.nlm.nih.gov/geo/

^2^ https://www.gsea-msigdb.org/gsea/msigdb/index.jsp

^3^ https://string-db.org/

^4^ http://mirwalk.umm.uni-heidelberg.de/
